# Designing context-specific physical activity interventions for English primary schools: key learning from a four-month rapid ethnography

**DOI:** 10.1186/s12889-025-23682-4

**Published:** 2025-07-18

**Authors:** Robert Walker, Danielle House, Simona Kent-Saisch, Alice Porter, Ruth Salway, Lydia Emm-Collison, Michael Beets, David Revalds Lubans, Frank de Vocht, Russell Jago

**Affiliations:** 1 Centre for Public Health, Population Health Sciences, Bristol Medical School, University of Bristol, Bristol, UK; 2 National Institute for Health and Care Research Bristol Biomedical Research Centre, University Hospitals Bristol and Weston NHS Foundation Trust and University of Bristol, Bristol, UK; 3 Centre for Exercise, Nutrition & Health Sciences, School for Policy Studies, University of Bristol, Bristol, UK; 4 Arnold School of Public Health, University of South Carolina, Columbia, SC USA; 5 Centre for Active Living and Learning, College of Human and Social Futures, University of Newcastle, Callaghan, NSW Australia; 6 Hunter Medical Research Institute, Newcastle, NSW Australia; 7 Faculty of Sport and Health Sciences, University of Jyväskylä, Jyväskylä, Finland; 8 National Institute for Health and Care Research, Applied Research Collaboration West (NIHR ARC West), University Hospitals Bristol and Weston NHS Foundation Trust and University of Bristol, Bristol, UK

**Keywords:** Physical activity, Children, School-based, Primary schools, Context, Tailored-intervention, Ethnography

## Abstract

**Background:**

Physical activity is essential for children’s health. Primary schools offer an opportunity to equitably promote physical activity. However, school-based interventions have been shown to have little to no effect, potentially due to a lack of consideration of school heterogeneity. This study reports on a rapid ethnography study that was used to capture insights into English primary schools physical activity. The data are intended to inform the design of a context-specific intervention to improve pupil physical activity.

**Methods:**

Three researchers conducted a four-month rapid ethnography study within three primary schools in Bristol, UK, between March and July 2024. Several methods were used: observations (*n* = 80), interviews (*n* = 26), photo elicitation with pupils (*n* = 4 activities, total 22 pupils), collection of documentary data (i.e. pupil demographics, school policies, etc.), informal conversations, and field notes. Reflexive thematic analysis was used to analyse the data.

**Results:**

Each school measured success in physical activity differently, such as increased opportunities, personal development, or broader curriculum attainment. Across all schools levels of pupil physical activity varied across physical activity opportunities in the school day, with breaktimes most active, PE lessons focused on fundamental skills, and active clubs providing quality but not fully inclusive opportunities. Furthermore, across all schools different school communities consistently had different goals and needs for physical activity: senior leaders were focused on how physical activity can support broader school-level strategies (e.g. academic achievement and student wellbeing); teachers were concerned with how physical activity can fit in and around curriculum pressures; and pupils wanted fun and engaging activities. Not all physical activities were feasible across settings, emphasising the need for tailored strategies. And differences in Parent Teacher Association (PTA) funding impacted resources and opportunities for pupil physical activity. These various areas of convergence and difference across the schools suggest strategies for intervention development.

**Conclusion:**

This study underscores the importance of context-specific approaches to promoting physical activity in primary schools. Context-specific intervention design should closely consider school context to ensure strategies are appropriate. Intervention designs should also include steps to understand different stakeholder goals, PTA funding disparities, and the appropriate areas of physical activity to target.

**Supplementary Information:**

The online version contains supplementary material available at 10.1186/s12889-025-23682-4.

## Introduction

Physical activity is important for children’s current and future health and wellbeing [1–3]. Both the UK Chief Medical Officer [4] and the World Health Organization [5] recommend that children accumulate an average of at least one hour of moderate-to-vigorous intensity physical activity (MVPA) daily. However, in the UK, only 41% of children aged 10–11 meet recommended activity levels [6].

State primary schools (children aged 4–11) provide a unique opportunity to equitably promote physical activity [7, 8]. In a recent scoping review of European primary school physical activity interventions, we identified 11 target opportunities across the school day (physical education (PE), active and outdoor learning, active breaks, breaktime, active play, daily movement initiatives, active travel, before/after school clubs, active homework, school-level environment, and community), with PE, physically active and outdoor learning (i.e. integrating physical activity into other key learning), and active breaks being the most commonly targeted [9]. However, most school-based physical activity interventions have either not been effective or only resulted in small improvements in physical activity [10–12]. We have argued that one potential reason for this is that most interventions adopt a “one size fits all” approach without considering important contextual factors such as organisational, political, cultural, and sociodemographic characteristics that vary between schools [13].

This study was conducted as part of the Physical Activity via a School-Specific PORTfolio (PASSPORT) study that is developing a context-specific approach to enhance school-based physical activity by tailoring interventions to each school. In PASSPORT schools will evaluate their context and select interventions that align with their needs, with the goal of achieving a more sustainable and successful physical activity intervention. In this present study we conducted a rapid ethnography to deepen our understanding of primary school context and how this impacts on pupil’s physical activity, to inform the development of our context-specific intervention.

Public health interventions must be suitable to the context they are designed for to be successful [15, 16]. Furthermore, understanding intervention context is essential to capture complexity and design complex interventions [17]. In a school setting this might include ensuring an intervention is manageable for busy school staff, supports their current systems, and is appropriate for their specific pupils. Ethnographic approaches have the potential to address these challenges by immersing researchers in the intervention setting, and combining observational, conversational, and documentary data collection. Traditionally these can be long research projects where research questions emerge through the process, however, both rapid and focused ethnography have evolved to adapt the principles of ethnography (combination of methods with immersion in a setting) within more bounded timeframes and applications [18–24]. Ethnographic approaches have been used to explore some elements of physical activity in schools, such as outdoor classrooms [25], PE [26], and after-school care [19], and rapid and focused ethnographies have been used to evaluate public health interventions [19, 21, 22, 27], but are less common in intervention development and are relatively rare within physical activity research. This rapid ethnography offers a novel perspective that may help optimise the design, feasibility, and implementation of context-specific physical activity interventions. This paper reports on key learning from a four-month rapid ethnography for designing context-specific physical activity interventions in English primary schools.

## Methods

### Participants and procedure

Over a roughly four-month period (between March and July 2024), we undertook observation, conversation, and documentary data collection related to pupils physical activity in three primary schools in Bristol, UK. This study was limited to four months due to project timeframes and the need to use this data and insight to inform the next steps of the PASSPORT study, and also due to school acceptability of the study. This approach provided a comprehensive understanding of structures, decision-making processes, and physical activity culture within each school. Through this study we captured multiple perspectives from the broad school communities over time, which enabled comparison across the schools, roles, and pupils.

We purposively sampled three schools to participate in this study and sought to include different school demographics and physical activity and PE structures to enable rich comparison across the schools. Indicators of these are described below. Two socioeconomic indicators informed our recruitment: (1) the percentage of pupils in the school eligible for free school meals (FSM) [28], a UK government scheme to provide free meals at school to children from low-income families [29], and (2) school postcode Index of Multiple Deprivation (IMD) decile, a measure of area deprivation [30]. The percentage of people who identify as Black, Asian and minority ethnic (BAME) in the school Lower Layer Super Output Area (LSOA, a UK statistical geography [31]) was also considered to capture diverse cultural and religious school contexts [31]. To understand the role and structures of physical activity in these schools, we drew on interview data from a related study which a staff member from each school had participated in [32, 33]. This information was used as an indicator of how physical activity was prioritised and organised in each school, for example, their reflections on school physical activity culture, information on who delivers PE and physical activity (i.e. a dedicated PE teacher, a class teacher as a PE lead, or other), as well as a sense of the facilities and space for physical activity each school had access to, in order to include diversity regarding attitudes and experiences relating to delivering physical activity.

School 1 was situated in an inner city neighbourhood on a relatively contained site. They have higher than average levels of socioeconomic disadvantage and BAME pupils, with a large number with English as an additional language. In our previous interviews, the school did not describe physical activity to be a top priority, however a dedicated PE teacher had recently been employed when this study began. School 2 had very high levels of socioeconomic disadvantage and a low number of BAME pupils. The school was located on the periphery of the city with a large site and grounds, and in our interviews the staff described sport and physical activity as a strong priority in the school and something they prided themselves on. Class teachers took on the role of PE lead. School 3 was located in a central neighbourhood of the city with a constrained site and facilities. They had a wide range of pupil socioeconomic position, with average proportions of BAME pupils. PE and physical activity were not the top priority of the school but they employed a dedicated PE teacher and strived to maximise what they could provide.

Participants within the schools included a range of school staff that have a role in delivering physical activity or broader health programmes, or in school governance and decision-making. Participants also included broader school community members who had a role in pupil physical activity or school governance, such as external providers of active clubs and PE, school Governors, or PTA members. Across this work and members of staff we focused on Key Stage 2 pupils and classes (Years 3–6, ages 7–11), and predominantly Year 5 pupils (aged 9–10), because the PASSPORT intervention will target this age group.

### Data collection

**Fig. 1:**
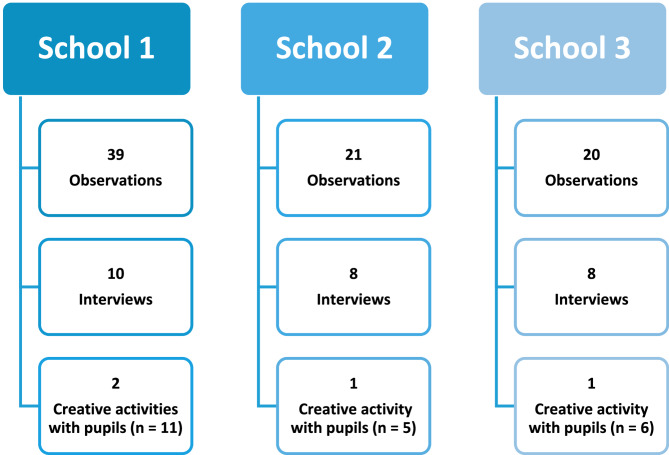
Data collection overview

We used a combination of six methods that covered observational, conversational, and documentary data collection, each described below including participant descriptions. An overview of data collected within each school can be seen in Fig. 1. DH led data collection in School 1, RW led data collection in Schools 2 and 3, and SKS supported data collection across all three, however. all researchers visited all schools for comparison and contextualisation. A study protocol and all data collections templates and materials are available on the Open Science Framework (10.17605/OSF.IO/8HE7A) [14].

#### Documentary data

Information on pupil demographics, physical activity programmes, funding, policies, and active clubs were collected via standardised forms completed by the research team, with staff input.

#### Observations

We conducted observations throughout the school day with a focus on occasions when pupils might be physically active (e.g. active travel outside the school gates, breaktimes, active clubs), but also non-PE lessons, wrap-around care (e.g. breakfast club), and lunch halls, to examine when, where, and how pupils were active. In addition, we observed school meetings and events (e.g. staff, Governor, and Parent Teacher Association meetings) to understand broader school structures and priorities. Where possible, activities were observed multiple times. A summary of observations across each school can be found in Table 1. Observations were recorded by completing templates to promote quality and consistency, prompting us to consider factors such as the number of children engaged in an activity. Researchers completed these at the nearest opportunity following the observation.

**Table 1:** Summary of observations by school

Observations	S1 *n*	S2 *n*	S3 *n*	Total obs *n*
PE led by external provider	3	2	N/A*	5
PE led by PE teacher	3	N/A	4	7
PE led by class teacher	4	3	N/A	7
KS2 non-PE lesson	3	2	6	11
Active club led by external provider	3	3	N/A	6
Active clubs led by school staff	2	1	N/A	3
Active/outdoor lessons (e.g. forest school)	2	N/A	0	2
Movement breaks	1	1	0	2
Lunch/break times in playgrounds/outdoor spaces	4	4	5	13
Sports event/competition	2	0	1	3
Active travel (AM)	1	1	1	3
Active travel (PM)	2	1	1	4
Wrap-around care session (AM)	1	N/A	N/A	1
Wrap-around care session (PM)	2	N/A	N/A	2
Student council/committee	1	1	0	2
Staff meeting	2	2	1	5
Governors meeting	1	0	0	1
Parent Teacher Association meetings	0	0	1	1
Healthy school or other award assessments	1	0	0	1
Healthy school or other award training	1	0	0	1
Total school n	39	21	20	80

* Not applicable, that activity was not provided at the school

#### Interviews

We conducted 26 one-to-one semi-structured interviews with school staff and community members to explore their perspectives on physical activity, school organisation, and structures. Interviews were conducted after several weeks of observation to ensure observations informed discussions. The guide was semi-structured, with prompts added based on the participant’s role and our data collection within the school, but broadly explored their responsibilities and experiences related to pupil physical activity. This included facilitators and challenges, decision-making and processes, and their experience of school policies.

Interviews were conducted by RW, DH, SKS, and AP and lasted from 23 min to 64 min (Mean = 41 min). After each interview researchers completed a reflection template to consolidate initial analytical ideas and next data collection steps. Interviews were transcribed verbatim using a university-approved service. A summary of interview participants across the schools can be found in Table 2.

**Table 2:** Summary of interview participants by school

Job role	S1 *n*	S2 *n*	S3 *n*	Total role *n*
Headteacher	1	0	1	2
Deputy headteacher	0	1	0	1
Business manager	1	0	0	1
PE teacher/leads	2	1	1	4
Relevant programme leads (e, g, active travel)	1	1	0	2
Key Stage 2 class teachers	1	1	2	4
External active club/PE lesson providers	1	1	1	3
PTA members	1	0	1	2
School Governors	1	1	0	2
Special Educational Needs Coordinator	0	1	0	1
Learning Support Assistants	1	1	1	3
Pastoral care	0	0	1	1
Total school n	10	8	8	26

#### Photo-elicitation with pupils

Pupils’ views were gathered using photo-elicitation with groups of up to 6 Year 5 pupils [34–36]. The pupils showed researchers around their school site in groups of two or three while taking photographs of places they thought were active or sedentary spaces. The photographs were then discussed with the wider group. This method allowed pupils to express their experiences with physical activity while providing time for relationship building between researchers and pupils in an engaging, hands-on way, which promoted richer discussions. We worked with their class teacher who identified pupils with diverse ethnic, socioeconomic, special education needs, and levels of engagement in school physical activity, who were then invited to participate. Group conversations were audio recorded and transcribed. Reflection templates were completed by researchers to capture reflections on the walks and the discussions that took place, and general impressions of the activity. An overview of activities and participants in each school can be found in Figure 1, and example photographs from each school are provided in supplementary file 1.

#### Informal conversations

Informal conversation with school staff and broader community members took place throughout the study, for example in the staff room or between meetings, and served to build our relationships, deepen our understanding of physical activity in the schools, and guided our next steps in data collection.

#### Field notes

Field notes supported all data collection, used to document useful information as well as reflections and analytical memos capturing interpretations and insights as they emerged [37, 38]. Field notes were written after each school visit to reflect on and consolidate learning, insights, analysis, and next steps. The field notes were digitised for easy sharing among the team, allowing for efficient searches of key terms, activities, and dates [39].

### Data analysis

We employed a reflexive thematic analysis [40] to interpret data, which was supported by weekly team discussions throughout the study where researchers reflected on their thoughts and perspectives. Data analysed included interview transcripts, photo-elicitation transcripts, observations, field notes, and interview and photo-elicitation reflections. NVivo version 1.7.1 was used to assist with the analysis [41]. The thematic analysis followed three key stages:

1. Data familiarization
  Through collective reflective processes built into the data collection, RW, SKS, and DH familiarised themselves with the dataset.
2. Coding
  To enhance reflexivity and deepen understanding, two interview transcripts, two observations, one day of field notes from each researcher, and a photo-elicitation reflection document were independently coded by RW, DH, and SKS, who then met to discuss their codes and interpretations. Codes were developed inductively to capture a range of descriptive, interpretive, and conceptual insights, encompassing both semantic (i.e. one that participants explicitly express) and latent elements (i.e. one that captures underlying ideas, meaning, or patterns). To manage the coding process across two researchers (RW and SKS) and the large dataset, we developed a shared codebook which evolved as the entire dataset was coded. This codebook was not developed with a positivist frame to establish consensus on meaning and eliminate researcher subjectivity, rather, it was a practical tool to sort and organise the data and enabled deeper researcher reflection and nuance. The codebook was generated iteratively to ensure codes were best suited for supporting theme development. RW and SKS met weekly to discuss changes or additions to the codebook and interpretations of the data.
3. Theme generation
  RW developed the themes for this analysis. Themes were defined as shared patterns of meaning across the data, each organised around a central concept, whether semantic or latent [40]. Theme development was initiated during the coding process and continued after coding was complete, considering the broader data set. Theme names were generated by RW through the process of identifying patterns of meaning, and all authors had the opportunity to adjust these to best reflect the data.

### Ethics and consent

At a school level, headteachers signed a school study agreement for the research to take place, including the collection of the school documentary data. Each school received a £500 payment as recompense for the time invested in supporting the study. A general study information sheet was provided to all school staff and community members. For observations, informed verbal consent was gained from the session leader, for example, the class teacher, and an observation information sheet provided. Interview information sheets were given to interviewees and written informed consent was gained digitally. Interview participants who were not on the school payroll (e.g. PTA members) received a £25 voucher as recompense for their time. For the photo-elicitation activity, parents and pupils received information sheets and parents provided informed consent online while pupils provided informed assent via a paper form [42]. Children who participated in the activity received a small gift (ball/frisbee toy) as a token of our appreciation.

This study was performed in accordance with the Declaration of Helsinki and ethical approval was gained from the University of Bristol, Faculty of Health Science Research Ethics Committee (FREC Ref 16095). All study methods were performed in accordance with approved ethical guidelines and regulations throughout.

## Results

Five themes were generated related to key learning for designing physical activity interventions for primary schools that centre school context. These were: (1) each school has their own measure of success in physical activity; (2) levels of physical activity vary across opportunities in the school day; (3) different members of the school community have different goals and needs for physical activity; (4) not all types of physical activity are feasible in every school; and (5) a PTA can provide significant physical activity resources. Themes 1, 4, and 5 demonstrate key differences across primary schools that can influence pupil physical activity, and themes 2 and 3 highlight areas of convergence.

Data excerpts are provided throughout to evidence key points within each theme. To improve understanding of the type of data, each excerpt is accompanied by a code consisting of a school indicator (e.g. S2 for School 2), followed by a letter indicating the type (observation (O), interview (I), or photo-elicitation activity (A)) and the chronological number it is associated with in the data set.

### Theme 1: each school has their own measure of success in physical activity

We observed little to no school-level policy or standardised assessment for physical activity outcomes, with schools being largely autonomous in their provision. As a result, schools measured their success in physical activity in different ways, reflecting their communities and priorities. By success in physical activity we mean how each individual school determined whether or not they had accomplished their individual aims and goals for their physical activity provision.

In School 1, physical activity was largely used to promote socioemotional well-being among pupils, many of whom experienced high levels of hardship. However, engaging pupils in physical activity, particularly PE, was very challenging due to these increased needs. As a result, improved attitudes towards and engaging children in physical activity became a key measure of success at this school. This may be reflected in their recent employment of a dedicated PE teacher to improve the quality and, therefore, engagement of pupils. As this school’s PE teacher noted:

“we know that some children have all those experiences and some don’t, so we want to try and level the playing field as much as possible” (S1-I4, head teacher).

“the football club has been running, that’s been really good to see as well because you can see them, week on week… the passion for football is really coming through. I think there’s been an improvement, as well, in terms of attitudes towards PE since starting”. (S1-I1, dedicated PE teacher)

This was similar within School 2, also a socioeconomically disadvantaged school. However, due to its location on the periphery of the city, providing opportunities that pupils did not have access to in their community was a key measure of their success. This could be seen in the school’s bi-monthly ‘enrichment’ activities that aimed to provide pupils with a wide variety of sports. Staff explained:

“…there isn’t as much going on, in terms of we don’t have any sports clubs around the area.” (S2-I3, class teacher).

“So, we’re at a stage now where we’re trying to offer children a variety of sports that they may not have played before… the children love it, and it just promotes sport and well-being. Hopefully, as well, some children will discover a sport that they’re really good at, that they may have not discovered otherwise.” (S2-I6, deputy headteacher).

School 3, a more affluent school compared to the other two schools, generally appeared to have a high level of pupil engagement in physical activity, which we observed in their PE lessons, movement breaks, and breaktimes. However, due to their constrained site, the provision of a high-quality PE curriculum and forest school (a nature-based education delivery model), regular transport to offsite facilities, and engagement in active travel, were all important measures of their success in physical activity. This could be seen through their investment in the dedicated PE teacher and forest school lead, as well as their engagement in active travel programmes and awards through the local authority/government. This is illustrated in the following interview excerpt:

“we spend a lot of money on those coaches to go up to [forest school site]. And we feel that’s really important because there’s only so much we can do in that hall. So for us, that’s a really important part of the PE spend of that budget.” (S3-I7, dedicated PE teacher).

### Theme 2: levels of physical activity vary across opportunities in the school day

We undertook observations of physical activity across the school day within all three schools, including active travel, active clubs, movement breaks in class, physically active and outdoor learning, breaktimes, and PE. Although we observed children being active in these opportunities, the activity levels in each activity appeared to differ greatly in length, intensity, and engagement, and this was fairly consistent across the schools.

Pupils had one morning or afternoon break of around 15 min and one lunch break of around 45 min to an hour. We observed that break and lunch times were noticeably the most active periods of the school day for pupils, with the majority of children running around and engaging in play or sport, without notable differences by gender. Break and lunch times were the only unstructured periods of the school day, with children free to choose their activity, which included imaginative/creative play, unorganised and organised sport or games (facilitated by staff or external providers), and dance. This may, in part, explain why children appeared to enjoy this active period of the day, which was clearly explained in the photo-elicitation activities with children, and shown in the following observations:

“[At breaktime] I feel relaxed and also I feel energetic and like I’m having a really good time.”

“at breaktime we’re all active rather than just sitting around” (both from S1-A1 transcript).

“Lunchtime looked very active with 95% [estimated] of children running around or playing. There didn’t seem to be a divide by gender and both girls and boys were playing” (S2-O1, lunchtime observation)

Following the PE curriculum, PE lessons focused on providing children with the fundamental movement skills needed to be active over their life course rather than in-session MVPA. As was noted in observations:

“The session wasn’t active, with the focus being on movement skills, apart from the 15 minute tag game at the end.” (S2-O3, PE observation).

“The activity levels feel generally quite low, with only some children looking to be out of breath and sweaty. Many of the skills required slower movements and seemed to aim at developing their physical literacy or movement skills.” (S3-O18, PE observation).

Active after-school clubs were also very active parts of the school day. However, participation in these was limited to smaller groups of children due to available spaces, cost, interests of the children, and parental support. Clubs, therefore, did not appear to reach as broad a range of pupils as breaktime.

“Because the session was so heavily based on games, many of which involved running around/tackling each other on the mats, the session was very active. The children were visibly warm and sweaty and were given water breaks around every 10–15 minutes.” (S2-O17, active club observation).

“So if they [pupils] need to go to a childminder or they need to go to after school club [wrap-around care that is not physical activity focussed] that’s where they need to be because actually a club that finishes at 4:00 is pretty inconvenient if your parents work.” (S1-I4, headteacher).

We observed that movement breaks and physically active learning were not often used to promote physical activity. Staff explained that it was very challenging to fit movement breaks into the pressured curriculum, even in School 1, which was equipped with Daily Mile (a national daily movement break initiative) playground markings that reduced the time needed to set up an activity. Movement breaks were, however, largely used in all schools as an intervention for pupils with Special Educational Needs and Disabilities (SEND) as a means of helping them to emotionally regulate, rather than to increase physical activity levels. As our interviews and notes on a pupil photo-elicitation activity illustrate:

“They [pupils] said they weren’t very active in their classroom, that they didn’t do movement breaks in the classroom like Just Dance. So they [pupils] took some photos of the desks and books […] and they decided to pose one of them as if they were reading a book.” (S1-A1 researcher reflections).

“Mornings are really hard, a) because they’re so tight for time because it doesn’t even feel like we have time to do a movement break… if there’s assembly and that overruns, and then the expectations for the curriculum are becoming so much more and you have to just ram them in, that there’s not really that time to have any of that physical break…” (S3-I2, class teacher).

### Theme 3: different members of the school community have different goals and needs for physical activity

Observations and interviews revealed the different goals and needs of school community members involved in pupil physical activity. Three school communities or groups were observed to have the greatest role in physical activity, namely, school senior leadership; teaching staff; and pupils.

Across the schools, the senior leadership teams (SLT) were focused on how physical activity could be used to promote the overall strategic vision of the school. SLT described the systemic pressures they are under, and core curriculum academic performance was a main priority, with physical activity playing a smaller role in supporting broader socioemotional skills and health. Therefore, the role of the SLT was to find ways in which stretched resources could be allocated to physical activity while promoting high levels of academic performance.

“…it isn’t clear as to exactly what you’re [the school] going to be judged on apart from exam results… and financial performance.” (S2-I5, school governor).

“to work towards an award of some sort requires time and capacity that everybody’s just always so stretched to do. So again, we’re like, “Why would we work towards the badge? What would that give us? What would that give our children?” […] when we’re saying no it is always about capacity, we’re not able to do it. And just I am really conscious that there is such a high workload with being a class based teacher […] so unless it can fit within what they’re [staff] being asked to do anyway [it can’t be prioritised].” (S1-I4, head teacher).

“As long as they can evidence they are doing PE and that, if Ofsted ever deep dive, it is seen to be good, the [school] Trust will leave it alone, letting the school decide their own curriculum. This is in stark comparison to core curriculum subjects that the trust seem to put a lot of pressure and expectation on schools to deliver very packed curriculums, trust-driven assessments, and interventions on core subjects that are lagging behind.” (Researcher reflections on S3-I4).

Teaching staff focused on delivering the strategic vision given to them by SLT. As in Theme 2, we observed the pressures facing teaching staff, who were being asked to deliver high volumes of work and physical activity must fit around it.

“as well as your basic English and maths, you have to teach your science, your PE, RE, geography, history, music, PSHC. Yeah, the list goes on, languages, design technology. You’re talking ten foundation subjects, and there’s five teaching days. So, it’s like how do you get that into the timetable, whilst ticking all the boxes? And sadly, it means that things like forest school falls off the timetable.” (S2-I6, class teacher and deputy head).

“The PE lesson was meant to start at 13:30 however the class was behind on spelling and reading which was prioritised.” (field notes on S1).

Naturally, however, pupils were not concerned with strategy or academic performance but rather driven by their own interests and having fun with the activities provided to them by staff. This was particularly apparent in physical activity. There were differences in the types of activity that children found engaging, most notably between younger and older children and girls and boys.

“Forest school is very fun. […] And at [external sports site] sometimes we do PE there, and that’s always fun.” (S3-A1, transcript).

“Year 5 had their [school Trust] sports day as well yesterday, which I think was a success. So those things have been really good. And I think children really love that time of year, when those things come around and there’s more of that happening.” (S1-I5, class teacher and previous PE lead).

### Theme 4: not all types of physical activity are feasible in every school

After observing physical activity within the three schools, it was apparent that not all types of physical activity opportunities or activities would be feasible in each. Reasons for this intersected around physical facilities and space, socioeconomic differences that impacted behaviour, staffing shortages, and daily pressures. For example, the limitations of School’s 1 and 3’s sites in urban areas meant certain PE lessons, active clubs, or forest school activities were limited or not possible. Both schools travelled off site for forest school, and School 3 was unable to put on any after school clubs due to space limitations.

“what I find really challenging about this site is the fact that you don’t have any leeway on the break times. You can’t go, “Oh, today, you can have five minutes extra playtime.” It just has to be on those confined times. There’s no flexibility in it.” (S3-I2, class teacher).

“[external provider] come in and do the after-school provision for childcare. [So] we don’t have somewhere where we can have a football club or whatever. […] I can’t run after-school clubs” (S3-I7, dedicated PE teacher).

Pupil behaviour presented school staff with significant challenges throughout the school day, and this impacted the physical activity they could deliver, while also faced with increasing pressures to ensure achievement in the core curriculum (Theme 3). Staff explained that coinciding with these pressures, the number of Learning Support Assistants and Teaching Assistants have reduced due to financial constraints, meaning that not every class had an additional adult to support learning or physical activity. Compared to the core curriculum, which usually takes place sitting in a calm and quiet atmosphere, physical activity is much less structured. As a result, we found that teachers’ willingness to engage in physical activities was reduced when a class had many children who struggled to regulate their emotions and behaviour.

“Behaviour in PE can be quite tricky because it has to be a lot more free.” (S1-I9, class teacher).

“there’s also the side of it that no matter what you want to do in terms of content, you will have the social interactions of that group playing a part. If they’re a harmonious cohort, there is so much more you can do but if they're not you also can’t ignore that. You have to approach them as a distinct social unit and sometimes edit or amend your plans so that you can get the best out of them and that they can have the most beneficial experience of sports” (S1-I3, external coach).

“…maybe, 10, 15 years ago, there would have been possibly one LSA, or one teaching assistant, in each class… In my opinion from budget cuts, and just the environment of schools, it’s very rare now that you have one adult allocated to a class.” (S1-I8, learning support assistant).

This particularly influenced the use of movement breaks. We observed that some class teachers were able to deliver a movement break for the class, allowing them to be autonomous, active, and have fun, confident that they would be able to easily reengage the class in their learning, whilst others experienced significant challenges in engaging children and movement breaks were seen as too disruptive.

“I was amazed by how calm and focused the class was. Because it was so clear the teacher was in control of the class, she could then have space to do other things– speak to me, prep the next task etc., but also allow them to go wild and have fun [with a video movement break], knowing it would be easy to bring them back.” (S3-O10, classroom observation).

### Theme 5: a PTA can provide significant physical activity resources

Primary schools in England face significant funding challenges. As a result, schools increasingly rely on additional sources of funding such as those raised by a Parent Teacher Association (PTA), if a school has one, to improve their school and support extracurricular activities. For example, interviews revealed that School 1’s PTA was in the process of raising £50,000 - £70,000 for new playground facilities and needed over £20,000 for new bike stores:

“But you know, just looking at those, the bike stores themselves aren’t that bad, £4,000 to £5,000 each, but we probably need four or five of them. So I’m talking about £20,000 or £25,000 It’s like, “Right, okay, I don’t have that money.” So it’s tricky. Yeah, bike stores is one, playgrounds is the other” (S1-I6, school business manager).

“… £50,000 to £70,000 for a playground will be their biggest goal so far.” (S1-I10, PTA member).

However, the effectiveness of each school’s PTA varied and was related to several factors. The first was having several motivated parent volunteers with the necessary skills to organise an effective PTA. For example, in School 3, a parent with corporate management experience led the PTA and organised activities that raised hundreds of pounds per week for the school, which was used to fund a term of forest school and outdoor clothes for the entire school.

“Some of the [PTA] meetings went on for hours and hours, with no real agenda… I think, for me, I just saw ways [the PTA] could build on its successes. [The PTA] has been really successful. But I was like, ‘actually, we could still do more’.” (S3-I1, PTA member).

The second factor included underlying socioeconomic factors within the school community. In schools based in areas with socioeconomic disadvantage, parents may not have the finances available to donate, regardless of how well-organised a PTA may be. In School 2, for example, the PTA could only fund small purchases, such as balls and other small equipment. Thus, these variations in PTA may further exacerbate socioeconomic physical activity inequalities.

“What I have got from them [the PTA], little bits for extra bits and bobs, footballs and stuff like that, they’ve been fine with giving me a few pounds. I don’t think their funding is massive. I don’t think they’ve got a massive amount, but a few quid they probably would give me.” (S2-I8, learning support assistant).

## Discussion

This study reports findings about the role of primary school context in pupil physical activity from a four-month rapid ethnography. This discussion will explore the key learning from this for designing context-specific physical activity interventions, and the novel insight of this approach.

In the primary/elementary school-based physical activity literature, there has been a shift towards more context-specific approaches, acknowledging the complexity and heterogeneity of school settings, including in England [43], Wales [44], and Germany [45]. Each of these approaches work closely with schools to understand their context and how physical activity can be feasibly promoted in each unique setting. Our findings further emphasise the importance of these approaches, highlighting areas of commonality across schools (Theme 2 on pupil activity levels during different activities, and Theme 3 on the priorities of different school communities) and areas of broad difference (Theme 1 on measures of success, Theme 4 on feasibility, and Theme 5 on the role of the PTA). This echoes the notion that despite some commonalities for schools within the same systems, in general school settings are highly diverse [13].

Schools know their specific context and needs, and this school knowledge needs to be harnessed by physical activity interventions. Although schools will likely need some culture change to better prioritise pupil physical activity, interventions that capture and understand the current culture and priorities of a school in advance of any intervention, and then seek to build on this, may lead to greater acceptance, suitability, and sustainability. For example, schools own measures of success in pupil physical activity that meet their specific communities needs (such as increased opportunities, personal development, or broader curriculum attainment), can be accommodated and supported by interventions, whilst identifying areas of school physical activity that can be developed. Key to this is an understanding of school physical activity culture. Although several studies have acknowledged the role of the social or cultural aspects of schools in physical activity [32, 33, 46, 47], and one systematic review has sought to understand what aspects of school culture play a role in physical activity [48], further research is needed to better understand what we mean by a physical activity culture in schools and how this affects pupils physical activity.

Our analysis highlighted three groups within school communities that have important roles in school physical activity yet have different needs and goals from it (the senior leadership team (SLT), teaching staff, and pupils). These needs and goals, however, appeared fairly consistent across the three schools, i.e. SLT needs, teaching staff needs, and pupil needs were broadly similar. Context-specific interventions that are designed based on only one of these stakeholder groups may lead to implementation, suitability, and sustainability challenges. For example, in English primary schools, teaching staff have repeatedly reported that increasing pressures and expectations are being placed on them [32, 49], with some describing their teaching workload as nearly unmanageable [32]. If context-specific interventions are not designed around this key challenge, teaching staff, who tend to deliver physical activity interventions, will likely struggle to implement the intervention. However, the convergence of goals and needs across these groups suggests a potential leverage for interventions to promote physical activity. For example, key SLT goals across the schools were academic performance, reporting to Ofsted, and pupil attendance. Designing interventions that understand these SLT priorities, such as interventions that align to Ofsted and other reporting structures, or using attainment and attendance as outcome measures, could improve buy in and build evidence for the role of physical activity in improving these outcomes.

This study demonstrated that not all physical activities or opportunities are feasible in all settings, due to intersections of a schools physical facilities and space, socioeconomic differences that impacted behaviour, staffing shortages, and other additional pressures, which is supported by other research [33]. Interventions may face limitations when based on identifying and attempting to implement physical activities which a school is not currently offering (e.g. if a school is not implementing movement breaks). There may be good reasons that schools are not currently providing certain physical activity opportunities. Researchers instead should work closely with schools to understand why certain activities are not being implemented, and then tailor interventions to accommodate any context-specific barriers in that setting.

This study found that levels of pupil MVPA varied greatly across the different physical activity opportunities in a school day. Our observations suggested that breaktimes were active for most pupils, which is reflected in other research [50]. Although this finding is positive in terms of children’s levels of physical activity, it is important to note that the length of breaktimes has been consistently declining for over 25 years in state schools in England [51]. Our observations of PE confirmed that PE provision was aligned to the national curriculum and focussed on developing skills and competencies in pupils, in a range of competitive and non-competitive physical activities [52, 53]. This aligns with research conducted in 2006, which identified that children spent 34% of the time in MVPA during PE, reflecting its educational focus where children were sedentary for long periods to listen to instructions, observe demonstrations, and organise lesson equipment [54]. Finally, we observed that active clubs provided excellent opportunities for MVPA for pupils, however participation in school-based clubs was uneven. These observations, combined with our other findings that highlighted areas of difference across schools physical activity, suggest that interventions that focus on one physical activity opportunity alone are unlikely to be sufficient. Whole-of-school physical activity approaches have recognised this and developed a body of work to engage the whole school system in promoting physical activity [43, 55, 56]. Additionally, a portfolio approach to interventions, combining several interventions across multiple opportunities in the school day and that are responsive to school context, might lead to an overall impact on MVPA [13].

Our previous research conducted in the same area of England as this rapid ethnography has shown that, although physical activity has, on average, returned to pre-pandemic levels [6, 57], how children are active has changed, widening certain socioeconomic physical activity inequalities [58–61]. Understanding socioeconomic inequalities is challenging, with school strategies usually focused on economic factors, such as providing free physical activity opportunities, which have reportedly had mixed efficacy [62]. Our findings related to the role of the PTA in socioeconomic inequalities may add useful insight towards this complex issue. This more hidden inequality may lead to higher frequency and quality of physical activity opportunities in more affluent schools, which in turn may provide children with the confidence and motivation to engage in wider, extra-curricular opportunities inside and outside the school. While clearly important and potentially very impactful, this more obscure part of the system that supports school physical activity is to a large extent beyond the control of schools and researchers. This therefore suggests the even greater importance of public health research to focus on equity and addressing inequalities within the immediate school system.

### Implications for practice

This study has five implications for designing context-specific physical activity interventions in primary schools, which can be seen in Table 3. Context-specific intervention design should (1) take steps to understand school culture and build on this, such as the schools’ specific measure of success in physical activity; (2) understand the different physical activity goals and needs of school communities and where possible align to these; (3) work closely with schools to develop strategies that are feasible in their specific context; (4) consider a portfolio approach that addresses physical activity across the school day; and (5) focus on addressing socioeconomic inequalities within the school system to mitigate against unequal sources of funding from the wider school community.

**Table 3:** Implications for practice

Key finding	Implications for practice
How schools measure success in physical activity varies between schools	Context-specific intervention design should seek to understand and then align with schools’ culture, such as their measure of success, and seek to build on these foundations.
Physical activity levels vary across physical activities and opportunities in the school day	A portfolio approach to interventions, that address physical activity across the school day, may have greater impact on physical activity levels.
Needs and goals for physical activity in school differ between school community groups	The different physical activity goals and needs of school communities should be considered in intervention design and where possible interventions should align to those priorities.
Not all types of physical activity are feasible in every school	Context-specific interventions should work closely with schools to understand their context and any barriers, in order to develop feasible strategies.
Parent Teacher Associations can provide significant physical activity resources	While an important factor of school physical activity, public health research and interventions must focus on addressing inequalities within the immediate school system, to mitigate the impact of this unequal external factor.

### Strengths and limitations

A key strength of this study lies in its ethnographic approach. To understand the complexities of primary schools, we have previously suggested a need to move beyond interview methods to locating researchers in schools [32]. Through a design that incorporates several data collection methods, multiple roles and perspectives within each school, and then enables comparison across schools, this study has provided a deep understanding of primary schools in England that can inform future context-specific physical activity interventions. Although this data is from a small number of schools in one part of the UK, these findings will be relevant to other countries as they provide insight into the complexity of school based decision making and competing priorities. This nuanced understanding may not be evidenced from more traditional qualitative approaches. Findings such as how schools have different measures of success in physical activity are hard to capture by other methods e.g. interviews or documentary sources alone. Schools’ measures of success were not written in policy but understood through the combination of observation and conversation over time in each school. An approach which enables researchers to be somewhat emersed in each school enables this nuance and these insights. However, there are several potential biases inherent in the approach that should be acknowledged. This study was limited to four months for data collection due to study and school constraints, and could have benefitted from longer engagement in each school to deepen understanding. Additionally, physical activity at schools is highly seasonal and, although we spanned half the school year (Terms 4, 5, and 6) and asked about other times of year, we may have missed information from certain periods in the school year.

## Conclusion

This study identified areas of convergence and difference in factors that influence pupil physical activity in primary schools, and the necessity for context-specific intervention design. This paper suggests context-specific intervention design considers a flexible approach that builds on each schools culture, understand the needs and goals of each school community, develops strategies that are feasible in that school, find approaches that work across the school day, and focus this work on equity and addressing socioeconomic inequalities within the school system.

## Supplementary Information

Supplementary Material 1

## Data Availability

As the PASSPORT project is still ongoing, data are not currently available. At the end of the project, data will be published as a restricted access dataset on the University of Bristol’s data repository (https://data.bris.ac.uk/data/) and access granted to approved researchers on request.
