# Supplementary material for: Designing context-specific physical activity interventions for English primary schools: key learning from a four-month rapid ethnography
Source: BMC Public Health. 2025 Jul 18;25:2497. doi: 10.1186/s12889-025-23682-4 (PMC12273025; doi:10.1186/s12889-025-23682-4)

Supplementary file 1: Example images from photo elicitation activities with Year 5 pupils

School 1

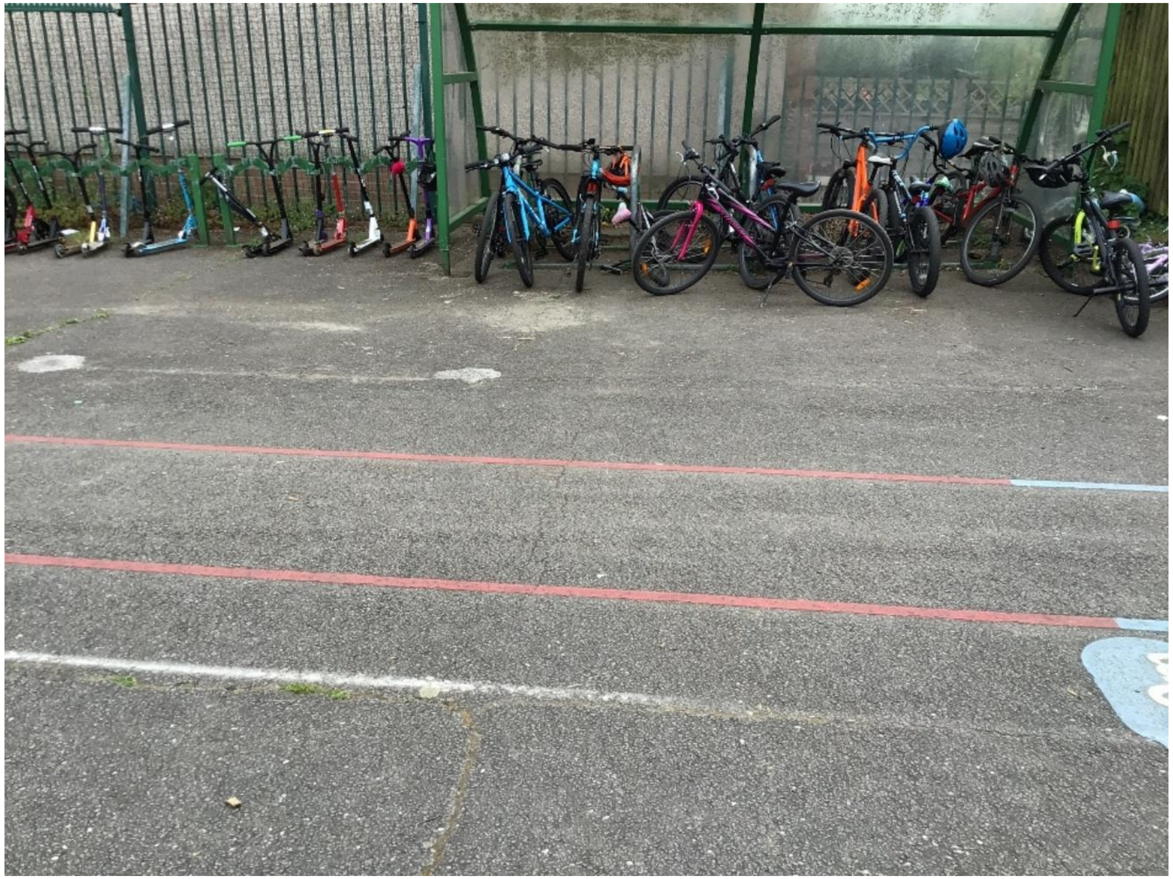

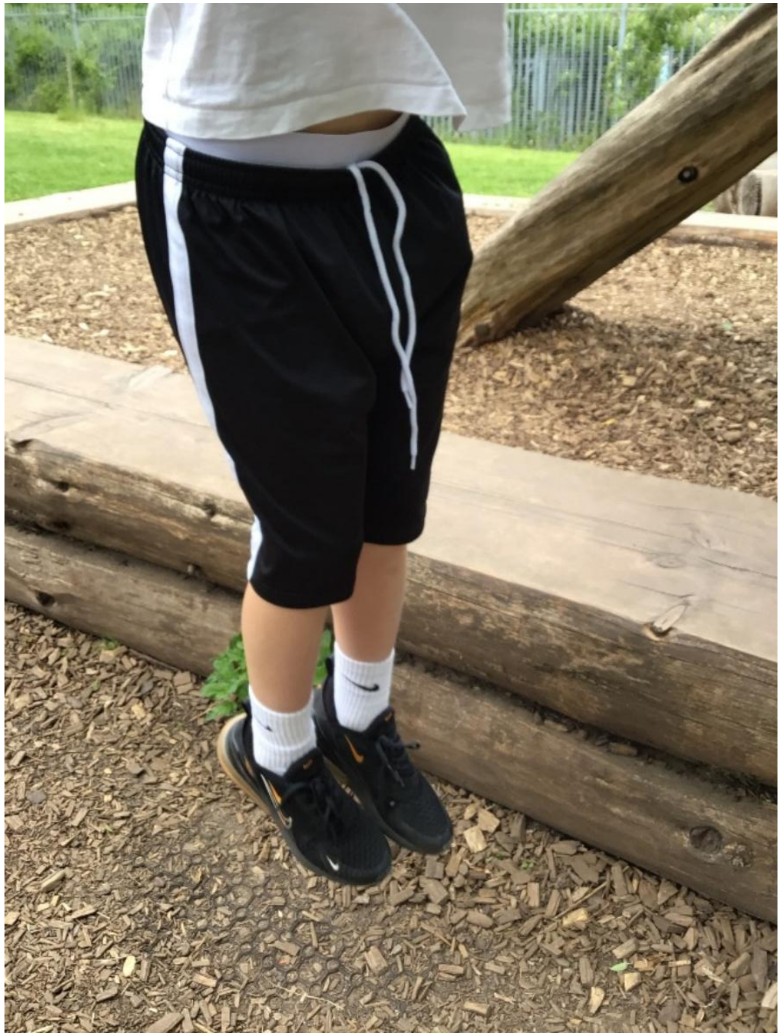

School 2

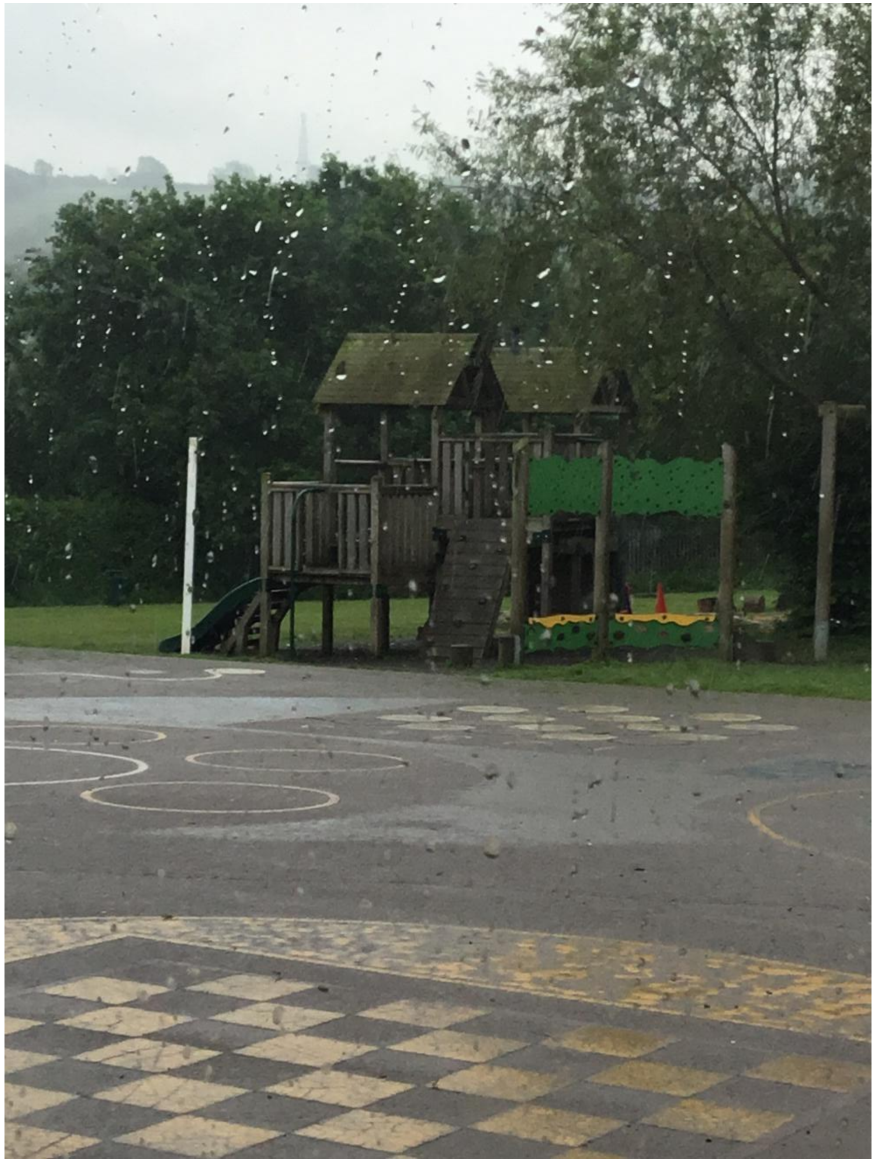

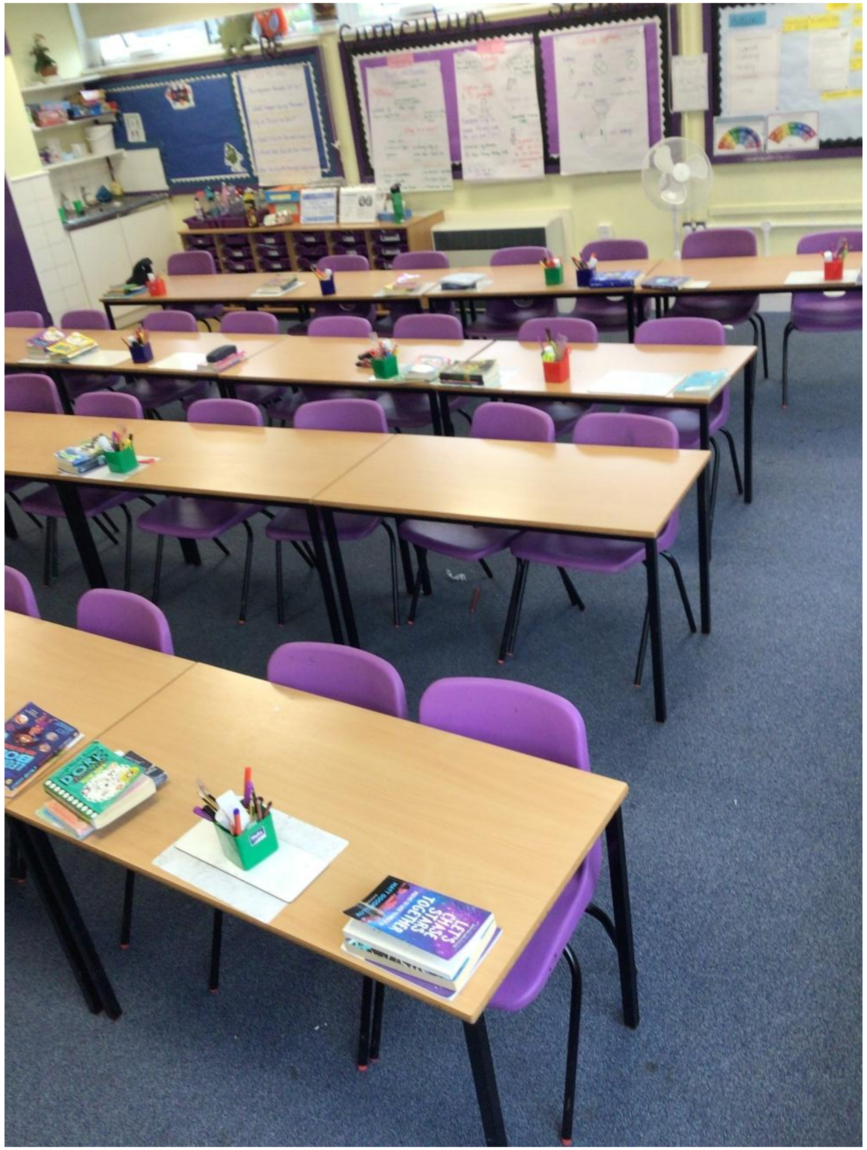

School 3

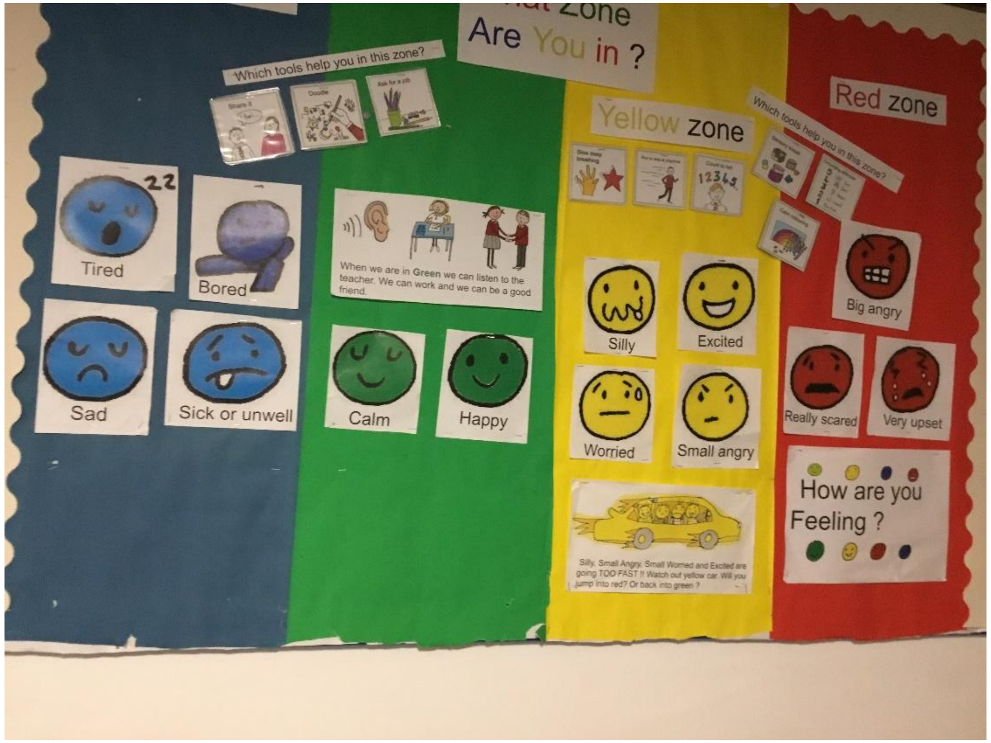

Are You in ?
Which tools help you in this zone?
Yellow zone
Red zone
Which tools help you in this zone?
Tired
Bored
Sad
Sick or unwell
When we are in Green we can listen to the teacher. We can work and we can be a good friend.
Calm
Happy
Silly
Excited
Worried
Small angry
Silly, Small Angry, Small Worried and Excited are going TOO FAST !! Watch out yellow car. Will you jump into red? Or back into green ?
Big angry
Really scared
Very upset
How are you Feeling ?

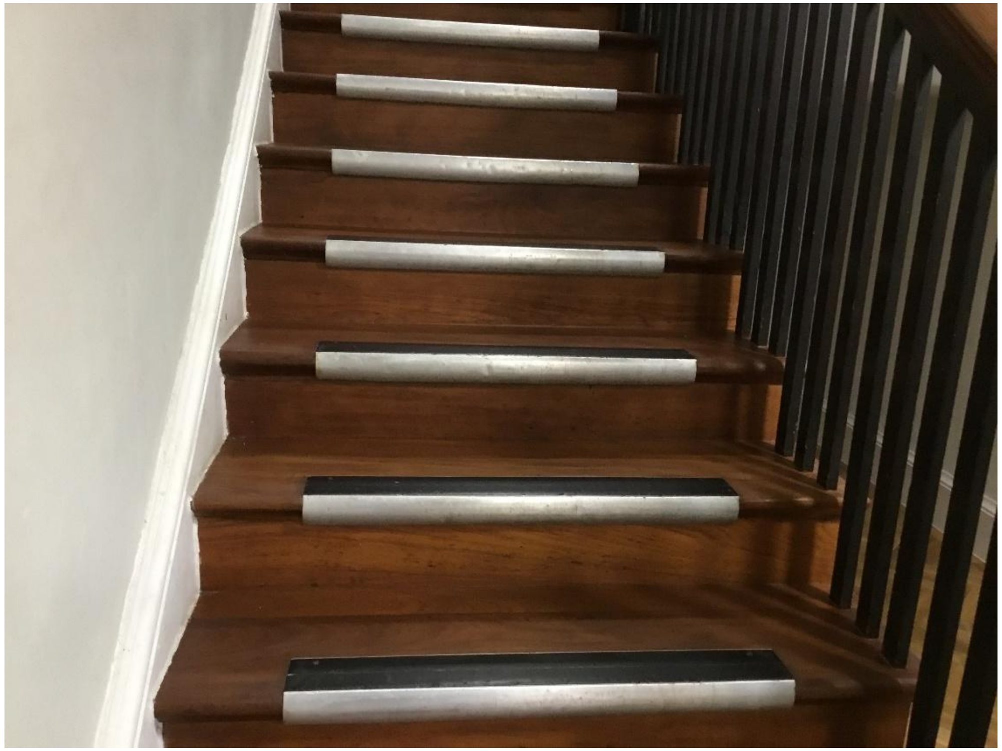

Supplement: Supplementary file 1 — Supplementary Material 1 [file 12889_2025_23682_MOESM1_ESM.pdf]
